# A deep learning mixed-data type approach for the classification of FHR signals

**DOI:** 10.3389/fbioe.2022.887549

**Published:** 2022-08-08

**Authors:** Edoardo Spairani, Beniamino Daniele, Maria Gabriella Signorini, Giovanni Magenes

**Affiliations:** ^1^ Department of Electrical, Computer and Biomedical Engineering, University of Pavia, Pavia, Italy; ^2^ Department of Electronics, Information and Bioengineering (DEIB), Politecnico Milano, Milano, Italy

**Keywords:** cardiotocography, deep learning, signal classification, signal processing, data science

## Abstract

The Cardiotocography (CTG) is a widely diffused monitoring practice, used in Ob-Gyn Clinic to assess the fetal well-being through the analysis of the Fetal Heart Rate (FHR) and the Uterine contraction signals. Due to the complex dynamics regulating the Fetal Heart Rate, a reliable visual interpretation of the signal is almost impossible and results in significant subjective inter and intra-observer variability. Also, the introduction of few parameters obtained from computer analysis did not solve the problem of a robust antenatal diagnosis. Hence, during the last decade, computer aided diagnosis systems, based on artificial intelligence (AI) machine learning techniques have been developed to assist medical decisions. The present work proposes a hybrid approach based on a neural architecture that receives heterogeneous data in input (a set of quantitative parameters and images) for classifying healthy and pathological fetuses. The quantitative regressors, which are known to represent different aspects of the correct development of the fetus, and thus are related to the fetal healthy status, are combined with features implicitly extracted from various representations of the FHR signal (images), in order to improve the classification performance. This is achieved by setting a neural model with two connected branches, consisting respectively of a Multi-Layer Perceptron (MLP) and a Convolutional Neural Network (CNN). The neural architecture was trained on a huge and balanced set of clinical data (14.000 CTG tracings, 7000 healthy and 7000 pathological) recorded during ambulatory non stress tests at the University Hospital Federico II, Napoli, Italy. After hyperparameters tuning and training, the neural network proposed has reached an overall accuracy of 80.1%, which is a promising result, as it has been obtained on a huge dataset.

## 1 Introduction

Nowadays, the health world is experiencing a never seen growth of collected data about patients and their clinical history, either those being acquired during care path but also during the entire span of their lifetime (Stead, 2018). Recent years saw the rise of new monitoring techniques, higher computational resources at lower costs, novel, and more powerful computational methods to extract parameters from more accurate and precise measurements. The presence of a large amount of new information generated at a consistent high rate, enforces the idea that medicine procedures could effectively improve their effectiveness by embedding the processing of such amount of recorded data (Hinton, 2018).

From a historical perspective, statistical analysis partially addressed this expectation by providing population classification and paving the way for the prediction of pathological events at least in terms of macro groups of subjects. The derived rules of inference were used to automate the medical reasoning process such as in the case of Linear Regression which represents a striking example of machine learning algorithm widely used in data analysis.

Machine learning (ML) methods can be considered a subset of AI techniques, characterized by the peculiar capacity to learn from huge amount of data as available for health-related applications. Such very large datasets are almost impossible to be analyzed by means of standard statistical methods. On the opposite, predictive models inspired by AI, can be learned on high dimensional datasets including many features (Naylor, 2018). Fetal monitoring during pregnancy by means of cardiotocography (CTG) and echography represents a good benchmark for ML methods, because it comprises a large amount of data coming both from signals and images.

In this paper we will focus on Fetal Heart Rate (FHR) signals, collected through CTG exams to perform a dichotomic classification (normal vs pathological fetuses) by means of a hybrid neural network architecture consisting of a Multilayer Perceptron (MLP) in parallel with a Convolutional Neural Network (CNN).

### 1.1 Brief history of CTG analysis

As a matter of fact, in developed countries, all mothers are submitted to medical examinations to monitor fetal wellbeing throughout pregnancy. Although most pregnancies proceed physiologically, complications affect approximately 8% of the total ones (4 Common Pregnancy Complications | John Hopkins Medicine, 2018). These might arise due to adverse mother’s health conditions, thus leading to various medical issues, further impacting the health of both the mother and fetus. The negative impact on the fetus health is usually referred to as “fetal distress”, which is strictly linked to alterations in the FHR signal.

The most employed diagnostic examination in the clinical practice is Cardiotocography (CTG). Such examination records simultaneously Fetal Heart Rate (FHR) and uterine contraction signals (TOCO). Conventional CTG started to be used since the 1970s as a non-invasive method to monitor fetal condition by eye inspection of both FHR and uterine contractions tracings (Hammacher and Werners, 1968).

The introduction of CTG considerably decreased fetal mortality during labor, but it did not improve the diagnostic performance as regards fetal morbidity, in particular during the antenatal period, mainly due to the qualitative examination of the tracings. The considerable inter and intra-observer variability and the inability of the human eye to extract quantitative information from the FHR signal played a key role representing the real weakness of the method (Ayres-de-Campos et al., 1999). Moreover, attempts made so far to interpret the tracings, according to various guidelines, did not provide the desired results (Ayres-de-Campos and Bernardes, 2010).

Analysis of CTG tracings received a boost since the early 1980s with the computerized CTG, which allowed to quantitatively reproduce the standard analysis method based on eye inspection of CTG signals time course (Visser et al., 1981). However, this was not enough to reach a satisfactory assessment of fetal wellbeing, although it eliminated intra and inter subject variability. The reliability of such approach has been limited for long time using basic time domain analysis, considering linear parameters only.

On the other hand, it has been observed that FHR changes anticipate and can predict fetal distress as well as adverse conditions before the insurgence of any other recognizable symptom (Hoyer et al., 2017). In this context, more sophisticated FHR Variability (FHRV) investigations have been proposed, stressing the importance of considering multiple parameters to assess fetal state (Van Geijn, 1996). Moreover, even frequency analysis parameters started to be used for quantifying fetal cardiovascular control mechanisms as it happened for adults (Malik et al., 1996).

A further development was introduced with the application of non-linear methods to biological time series, which can investigate the geometric and dynamic properties of the FHR signal. Entropy estimators (Pincus, 1991), complexity indices (Lempel and Ziv, 1976) as well as wavelets (Daubechies, 1990) and other nonlinear related parameters were applied with the aim to improve the information enhancement from the FHR (Pincus and Viscardo, 1992; Ferrario et al., 2007; Spilka et al., 2012). Such techniques allowed to describe and understand complex physiological control mechanisms thanks to novel available tools. A review of the most used nonlinear indices applied to FHR was recently published by Ribeiro et al. (Ribeiro and Montero-Santos, 2021). These nonlinear indices were added to more traditional signal processing parameters developed in time domain such as the ones derived from classical analysis in time domain: Short- and Long-Term Variability (STV and LTV), Delta and Interval Index (II) as proposed by Arduini et al. (Arduini and Rizzo, 1994).

### 1.2 Artificial intelligence in CTG analysis (machine & deep learning methods)

The multiparameter approach aroused great interest, in particular, in the evaluation of the onset of states of fetal pathology. However, as the number of parameters increased, even a multifactorial statistical analysis became very difficult to be applied and researchers in the field of fetal monitoring started to consider AI techniques. Several approaches have been proposed in the literature since the introduction of the computerized CTG analysis, which allows to quantify the FHR behavior by means of both linear and non-linear aspects. These aspects consider the indices used in traditional diagnostics, novel and advanced regressors coming from quantitative frequency analysis, nonlinear parameters, and are integrated with maternal information. The different sets of features are used for the classification of the occurrence of pathological states or simply for the assessment of the maintenance of the healthy condition. The results strongly depend on the number of cases, the used database, the considered features and the performance of the classifiers

Fergus et al. (Fergus and Hussain., 2017) utilized Machine Learning models to classify caesarean section and normal vaginal deliveries based on cardiotocographic traces. In this study 552 FHR signal recordings, of which 506 controls and 46 pathological, were used as dataset, from which features like baseline, accelerations, decelerations, Short-Term Variability (STV) and many others have been extracted. The models adopted in this paper are multi-Layer feedforward neural network, Fisher’s Linear Discriminant Analysis (FLDA) and Random Forests (RF).

These methods, based on predictive learning classifiers, are known to suffer from the limitation of relying on the extraction of complex hand-crafted features from the signals. Therefore, research in this field has been moving in the direction of deep learning techniques. Petrozziello et al. (Petroziello and Jordanov, 2018) make use of raw signals from Electronic Fetal Monitoring (EFM) to predict fetal distress. They fed a Long Short-Term Memory (LSTM) and a CNN network with both FHR and UC signals, reaching a predictive accuracy of respectively 61% and 68%. It is worthnoting that their dataset consisted of 35429 recordings, but contained 33959 healthy newborns, while only 1470 compromised, resulting to be strongly unbalanced.

Iraji et al. (Iraji, 2019) explored other soft computing techniques to predict fetal state using cardiotocographic recordings. Neuro-fuzzy inference system (MLA-ANFIS), Neural Networks and deep stacked sparse auto-encoders (DSSAEs) were implemented. Iraji used a limited dataset composed of 2126 selected recordings that were divided in three classes: 1655 normal, 295 suspect, and 176 pathologic. On the full dataset, the best performing approach was deep learning with an accuracy of 96.7%, followed by ANFIS that reaches an accuracy of 95.3%.

Zhao et al. (Zhao et al., 2019) used FHR signals transformed into images by using Continuous Wavelet Transform. Their models consist of an 8-layer Convolutional Neural Network (CNN) with a single Convolutional Layer. Their dataset was the open-access database (CTU-UHB), with 552 intrapartum.

FHR recordings, containing a noticeable percentage (about 20%) of scalp electrode recordings. Their model reaches a 98.34% of accuracy with an AUC of 97.82%.

More recently, Rahmayanti et al. (Rahmayanti et al., 2022) propose a comparison between ML methods for the classification of fetal well-being using 21 attributes from the measurement of FHR and UC. They report excellent levels of accuracy. The dataset used was obtained from the University of California Irvine Machine Learning Repository, which is a public dataset. It consisted of 2126 data on pregnant women who are in the third trimester of their pregnancy collected through the system Sys Porto. In their study, the application of deep learning methods did not produce satisfactory and improved results compared to the ML approach The authors consider that using a more representative db and perfecting the set of variables can improve performance.

The contribution of Su Liu et al. (Liu S et al., 2021) stresses the importance of integrating echo images with cardiotocographic data for improving the classification of fetal states. The goal of the study was to improve the feasibility and economic benefits of an artificial intelligence based medical system when Doppler ultrasound (DUS) imaging technology are combined with fetal heart detection to predict the fetal distress in pregnancy-induced hypertension (PIH).

Finally, the review by Ki Hoon Ahn, et al. (Ahn and Lee., 2022) presents a comprehensive overview of the possible application of AI, DL and ML in Obstetrics for the early diagnosis of various maternal-fetal conditions such as preterm birth and abnormal fetal growth. The paper purpose was to review recent advances on the application of artificial intelligence in this medical field. The paper summarizes in table form the main characteristics of the different studies using AI, ML, DL methods. From this work we understand the pervasiveness in the field of fetal medicine of AI methodologies. There is also a perception of the complexity of the work still to be done to build reliable and validated classification systems. The data shown shows the great variety in terms of applications and the number of data collected/used that define the final performance of the analysis tools.

As a general remark, it is possible to notice that the global accuracy and performance of AI methods for perinatal medicine so far published in the literature are almost inversely proportional to the number of cases: the best results are obtained with limited and selected datasets.

In fact, the main limitation imposed by deep learning techniques is the huge number of data needed to train the neural architectures. Hence, the use of an inadequate number of records could lead to an overestimation of the generalization capabilities of the model.

In the present work we propose a neural model with two branches consisting respectively of an MLP network and a Convolutional Neural Network (CNN). This neural architecture receives heterogeneous input data, i.e., a set of parameters and images. The aim is to exploit the neural network generalization capacity by integrating FHR quantitative regressors, known to summarize the pathophysiological condition of the fetus, (either in time, frequency, and non-linear domains) with some new features implicitly learned from images, consisting in various representations of the raw FHR signal (time-frequency, recurrent patterns, etc.). A further novelty in the field of fetal monitoring consists of using a dataset containing 14,000 real cardiotocographic entries, with a perfect balance between healthy and pathological subjects.

**FIGURE 1:**
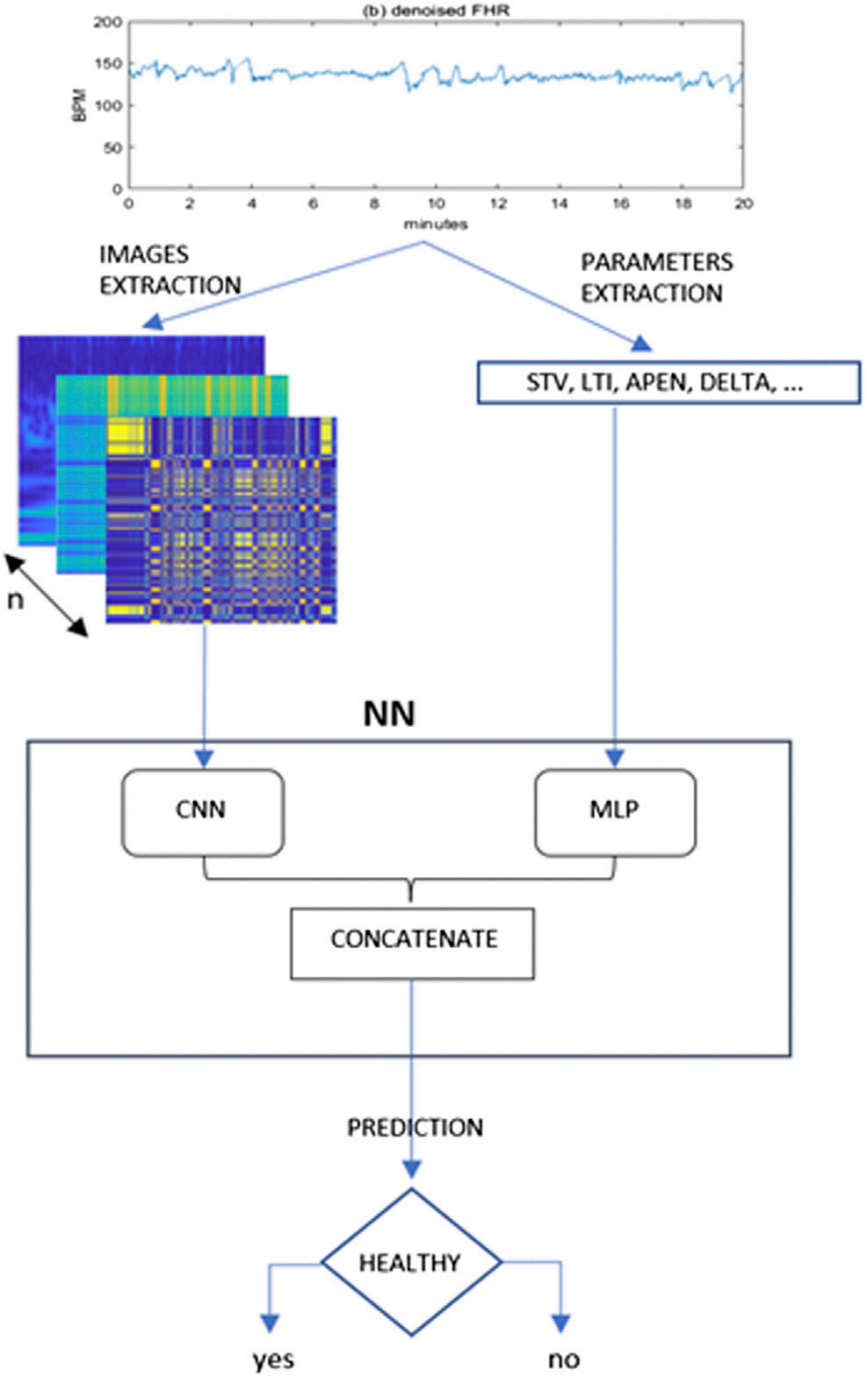
Proposed framework for the present study. From the denoised 20 min FHR sequence a set of images and quantitative parameters are extracted and fed to a CNN and MLP branch respectively. The outputs of these two typical neural networks are then concatenated in a single Mixed Type Neural Architecture for the classification task.

The framework proposed is depicted in Figure 1.

## 2 Methods

### 2.1 Database description and preparation

The FHR signals used in the present study were obtained from ambulatory CTG recordings collected from 2013 to 2021 at the ObGyn Department of the University Hospital Federico II, Napoli, Italy, during standard antepartum non-stress monitoring.

Each CTG exam was performed in a controlled clinical environment with the patient lying on an armchair. The CTG tracings were measured using Philips and Corometrics cardiotocographs, equipped with an ultrasound transducer and a transabdominal tocodynamometer. Raw signals were provided by the cardiotocographic devices to the 2CTG2 software (Magenes et al., 2007), which stores both the FHR and the Toco signals at a sampling frequency of 2 Hz. Therefore, every minute of recording consists of 120 points for each of the two signals. Each CTG exams lasted at least 60 min.

Each record was then classified and labelled by the medical team as healthy or belonging to a specific pathological group. Different numerical codes were associated to different antenatal pathologies, thus making easy to extract recordings belonging to the different categories from the whole DB.

The 2CTG2 software computes and automatically stores a set of regressors describing the statistical characteristics of the signals themselves, as described in (Magenes et al., 2007). The whole cohort consisted of 9476 pregnant women with a total number of 24095 CTG records.

The database was then cleaned and structured, by eliminating records without valid annotations and/or missed anamnesis. This procedure produced a clean version made of 17483 valid entries, with 7733 healthy and 9750 pathological tracings.

Pathological group included tracings of subjects with different diseases both of maternal and fetal origin, such as diabetes, malformations, intrauterine growth restriction (IUGR), etc.

As the goal of the study aimed at the separation between healthy and pathological fetuses, each entry of the dataset was binary categorized. Recordings belonging to the physiological pregnancy group (Normal) were denoted with 0 and those presenting a disease condition (Pathologic) were denoted with 1.

At this point, the FHR signals were submitted to a pre-processing procedure, as described in Section 2.2. On the basis of the FHR quality in each recording, a balanced set of 14000 tracings was selected (7000 normal and 7000 pathological). Thus, our final dataset contains a quite large number of fetal records, balanced by category.

These characteristics are fundamental as the training of most AI algorithms requires large balanced structured datasets to avoid polarized and inconsistent results which are both weaknesses affecting deep learning method applications.

### 2.2 Signal pre-processing

In clinical practice, the FHR signal is recorded using an ultrasound probe placed externally on the abdomen of the pregnant woman. Movements produced by the opening and closing of the fetal cardiac valves represent the information content of the United States signal. By an algorithm based on the autocorrelation function, the firmware of the CTG monitors reconstructs with good accuracy the occurrence of the fetal beats providing the FHR signal (Lawson and Belcher, 1983).

However, there are several factors that can affect the measurement of FHR, such as the movement of the mother and fetus, the displacement of the transducer till events in the external clinical environment. This can result in artifacts and signal losses which are the major sources of noise in the fetal signal. Therefore, the main goal of the pre-processing phase is to reduce these disturbances that worsen the successive phases of the analysis of the signals. Signal intervals with losses having a duration of less than 15 s, were removed through linear interpolation procedures. Losses of longer duration instead, were completely removed from the signal.

**FIGURE 2:**
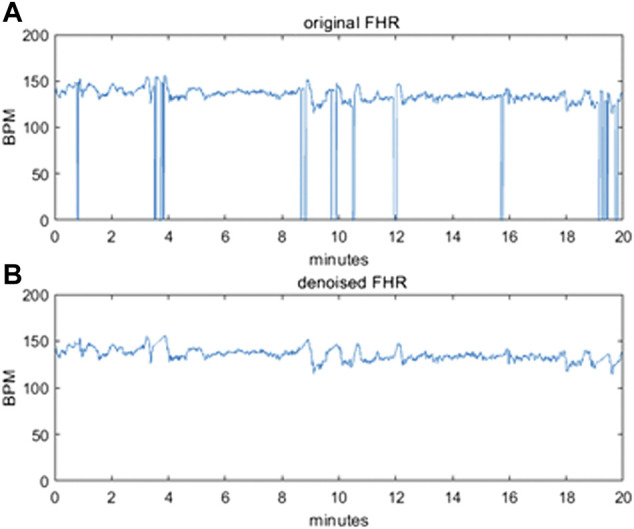
**(A)** original raw FHR signal before pre-processing **(B)** denoised FHR signal after pre-processing.

To avoid polarizations induced by the difference in the length of the FHR sequences, we decided to use sequences of exactly 20 min each, corresponding to 2400 points. Even in this subset, the intervals with an excessive level of corruption by noise and with signal loss were removed from the dataset. Figure 2 illustrates an example of a raw 20-min signal before correction (a) and after denoising (b).

Once clean recordings were available, the system was ready to extract the parameters of interest (detailed description in Section 2.3).

All the pre-processing steps have been implemented in MATLAB 2021a (The Math Works, Inc.).

### 2.3 Feature extraction

With the intent of feeding the MLP branch with a set of quantitative regressors describing the statistical characteristics of recorded signals, we considered a set of parameters for each of the 20 min split and processed FHR signals in our dataset.

Among the features calculated from the FHR signal, the subgroup that constitutes the selection of the ones included in the analysis, was made based on the literature study. This was followed by a process of feature selection and correlation analysis, starting from a wide group of more than 30 regressors, commonly used in fetal monitoring, and known to provide pathophysiological meaning related with the control mechanisms of heart. In particular, we decided to include in our study all the parameters evaluated in (Signorini and Pini, 2020), which provided good results in the classification of Normal and IUGR fetuses, although with a small dataset.

The final set consists of 15 quantitative parameters. The parameter set includes 4 linear parameters describing signals in time domain, namely: DELTA, Interval Index (II), Short-Term Variability (STV), Long-Term Irregularity (LTI) computed as described in (Arduini and Rizzo, 1994), 3 linear parameters related to frequency domain signal content i.e., Low Frequency (LF), Movement Frequency (MF), High Frequency (HF) as described in (Signorini et al., 2003) and the complex, non-linear parameter Approximate Entropy (ApEn) (Signorini et al., 2003). These parameters were automatically extracted by the 2CTG2 software. Moreover, we also included the FHRB that is the mean value of baseline, extracted with a modified version of Mantel’s Algorithm (Mantel and van Geijin, 1990), the ratio in the power spectrum bands (LF/(MF + HF), the number of small accelerations (>10 bpm and <15 bpm for 15 s), the number of large accelerations (>15 bpm for 15 s), the number of decelerations (>20 bpm for 30 s or >10 bpm for 60 s) (Arduini and Rizzo, 1994). Two more indices were considered as input values for the MLP branch: the Gestational Week and Mother’s age.

This parameter set covers most of the information the FHR signal contains as it takes into account time domain changes, frequency domain linear components and complexity signal characteristics associated to nonlinear dynamic evolution.

Since the considered parameters have different scales, before providing them as inputs to the MLP, we applied a normalization procedure by scaling all parameters in the range 0–1 using the min-max normalization.

### 2.4 From FHR signal to images

The major goal of our approach was to exploit the implicit ability of neural networks to learn complex features directly from the available data, without summarizing them by means of the statistical regressors described in Section 2.3. Thus, in parallel to the MLP branch we created a convolutional neural network (CNN) branch because CNNs have already shown great abilities in extracting important features from images and in image classification tasks (Krizhevsky et al., 2012).

Signal to image transformations are becoming more and more common since the recent successes got by deep learning in the field of computer vision.

Thus, we decided to encode the denoised FHR signals, obtained after preprocessing, into a set of images representing the FHR behavior by means of various computational transformations. In other words, the convolutional branch exploits the CNN’s ability to analyze two-dimensional objects (images) by encoding the FHR signal information content in a 2D domain.

**FIGURE 3:**
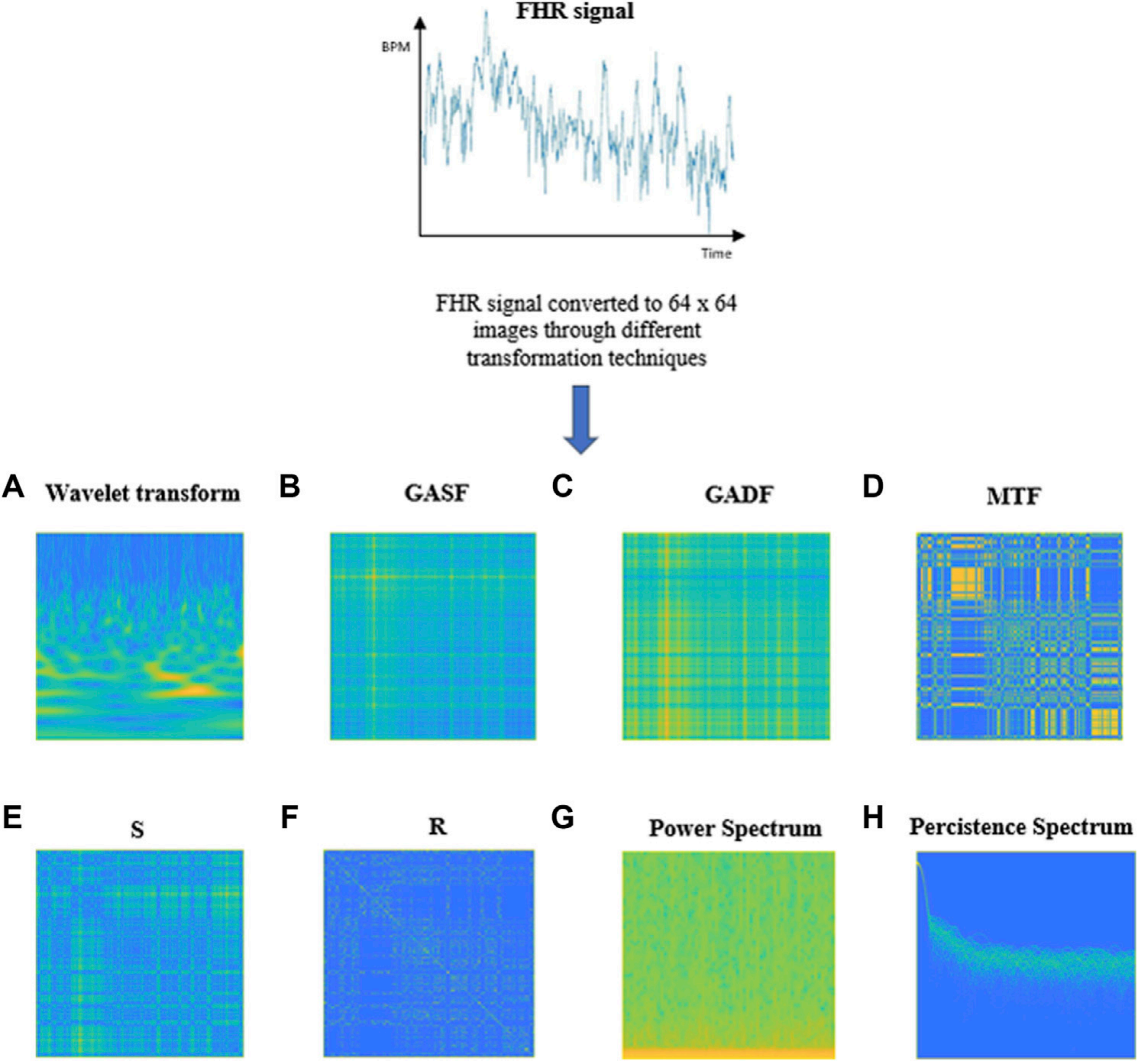
Examples of images obtained through the different transformations applied to a 20-min FHR signal. **(A)** Wavelet Transform, **(B)** GASF, **(C)** GADF, **(D)** Markov Transition Field, **(E)** S, **(F)** R, **(G)** Power Spectrogram, **(H)** Persistence Spectrum.

For our purpose we decided to use eight transformation techniques which are represented in Figure 3, with the same fictious “parula” colormap, and briefly described in the following subsections, to allow the system to automatically grasp different aspects about the nature of the FHR signal from the different images provided.

The choice of the particular set of techniques employed to encode the FHR signals into images has followed an in-depth literature search. Our intent was to exploit the intrinsic capacity of CNNs to automatically select the most relevant features, starting from the images provided as input. For that aim we selected a set of transformation techniques to obtain a group of images that could allow a description of the FHR signals, from different points of view, as much complete as possible. To provide a time-frequency view of FHR tracings, both spectrograms and scalograms were considered. Scalograms represent the analog to power spectrums when dealing with wavelet transforms; they generally provide a better time localization for rapid, high frequency events and a better frequency localization for low-frequency, longer-duration events. However, since the best time-frequency representation depends on the specific application, both spectrograms and scalograms were included in our study. The use of scalograms to encode FHR tracings into images, used as inputs for a CNN net, were already proposed in the work by (Zhao et al., 2019). However, their dataset was limited to 552 records (of which 447 normal and 105 pathological), so that a process of data augmentation was necessary to obtain a sufficient number of records for the training of the proposed neural model.

Moreover, persistence spectrums were included in our study, since they provide information about the persistence of a certain frequency in a signal during its evolution.

Together with the aforementioned techniques, which are used to obtain time-frequency representations of signals, other methos were employed to explore different aspects of FHR tracings, such as their evolutional dynamics. Among these, Markov Transition Fields were taken into account. The latter allow to obtain a visual representation of the transition probabilities, for each time point in the sequence, that maintains their sequentiality, in order to preserve information in the temporal dimension.

To explore the presence of recurrent patterns or irregular cyclicities in the FHR tracings, recurrence plots were also considered, as they provide visual representations that reveal all the times when the phase space trajectory of a dynamical system visits roughly the same area in the phase space.

Another transformation technique that we included in our work is Gramian Angular Field, which provides a description of the temporal correlation structure of a time series, through the use of a polar coordinate system.

Markov Transition Fields, Recurrence Plots and Gramian Angular Fields have already been employed as methods to transform time series into images, as illustrated, for example, in the work from (Wang and Oates, 2015). However, none of the studies in the literature reports the use of these techniques to encode FHR tracings.

The different transformations were applied to encode each 20 min of clean FHR signal into corresponding images, as illustrated from Section 2.4.1 to 2.4.6.

Even in this case, the images generated through the different methods were characterized by different scales and were so mapped in [0,1] range through min-max normalization. Moreover, all images so far obtained, were reduced in size to a dimension of 64 × 64 × 1.

Signals to images encodings were implemented by using the software MATLAB 2021a (The Math Works, Inc.).

#### 2.4.1 Continuous Wavelet Transform

The wavelet transform (WT) (Daubechies, 1990) is a mapping from L^2^(R) → L^2^ (R^2^), with superior time-frequency localization as compared to the Short Time Fourier Transform (STFT). This characteristic opens up the possibility of a multiresolution analysis.

WT has been extensively employed in biomedical engineering to analyze non-stationary and nonlinear signals over the last decades. CWT presents great abilities, such as its flexible capacity to extract general and fine-grained feature information from the input signal.

Continuous Wavelet Transform (CWT) is a formal tool that provides a hyper-complete representation of a signal by performing the convolution of a signal with a rapidly decaying oscillating finite-length, waveform, called mother wavelet, whose translation and scaling varies continuously.

The result of these convolutions is a series of coefficients, obtained for each time point, that are used to create a 2D representation of the signal, called scalogram. The *x*-axis coincides with the time axis and the *y*-axis with the scaling factor of the mother wavelet.

Each point of this 2D map represents the intensity of the corresponding (associated) coefficient and it is shown using a particular colormap.

More detailed information about the mathematics behind this approach can be found in Supplementary Appendix SA. The primary reason for applying the CWT in this research is that the CWT can provide a better method than others for observing and capturing the local characteristic information which is hidden in the FHR signal both in time and frequency domains.

An example of image obtained by applying the CWT to 20 min FHR signal of our DB is shown in Figure 3A. The yellow portions in the image represent the coefficients with the higher intensity values while the blue portions define the coefficients with the lower intensities.

#### 2.4.2 Gramian Angular Field

Gramian Angular Field (GAF) (Wang and Oates, 2015) generates an image, obtained from a time series, which shows the temporal correlations between each time point in the time signal. GAF images represent a time series in a polar coordinate system instead of the typical Cartesian coordinates.

GAF images depict the relationship between every point and each other in the time series, that is, it displays the temporal correlation structure in the series. The greatest advantage of GAF is that it can preserve temporal dependencies and leading to bijective encodings.

It is possible to obtain two different kinds of GAF, i.e., the Gramian Angular Difference Field (GADF) and the Gramian Angular Summation Field (GASF).

The details for obtaining GADF and GASF are illustrated in detail in Supplementary Appendix SB. Figure 3B and Figure 3C respectively report an example of a GASF and a GADF Gramian Angular Difference Field) images, obtained from an FHR signal of 20 min of our dataset.

#### 2.4.3 Markov Transition Field

Markov Transition Field (MTF) (Wang and Oates, 2015) provides an image which is obtained from a time series. The image contents represent a field of transition probabilities for a discretized time series. Given an n-length time series, MTF is a n x n matrix containing the probability of a one-step transition from the bin for x_k_ to the bin for x_l_, where x_k_ and x_l_ are two points in the time series at arbitrary time steps k and l.

To make the image size manageable and the computation more efficient, we reduced the MTF size by averaging the pixels in each non-overlapping m × m patch, that is we aggregate the transition probabilities in each subsequence of length m together.

More details about the construction of a MTF can be found in Supplementary Appendix SC. Figure 3D shows an example of an MTF image obtained from a 20 min FHR sequence belonging to the dataset used in our work.

### 2.5 Recurrence plot

Recurrence plots (RP) were introduced as a visualization tool to measure the time constancy of dynamical systems (Wang and Oates, 2015).

Natural processes can have distinct recurrent behaviors like periodicities (as seasonal cycles) or irregular cyclicities. A RP, generally defined as R, depicts all the time instants when the phase space trajectory of a dynamical system visits the same area in the phase space. A recurrence of a state at time i at a different time j is marked within a two-dimensional squared matrix where both axes represent time.

A RP can be generated by first computing a distance matrix S that contains each distance from one point in the time series with each other and then applying a threshold ε to binarize the values. An example of S and R images obtained from an FHR series included in our dataset is shown in Figure 3E, F, respectively.

More details about the construction of S and R can be found in Supplementary Appendix SD.

### 2.6 Power spectrogram

A power spectrogram (PS) (Sawa et al., 2021) is a visual representation of the frequency spectrum of a signal (*y*-axis) as it varies with time (*x*-axis). The most common way to show a spectrogram is using a heat map which uses a system of color-coding to represent different intensity values.

Given a time series, we can estimate spectrograms with methods based on Fourier transform (FT) or by using filter banks. Our choice was to adopt a FFT approach. This method splits data into chunks, which usually overlap, and proceed to compute the Fourier transform of each chunk to calculate the relating frequency spectrum magnitude. Each vertical line in the image corresponds to a chunk, a measurement of magnitude versus frequency for a specific moment in time. These so-called spectra are then put sequentially to form the image. Hence, given a time series s(t), to retrieve the image we need to apply a short-time Fourier transform (STFT) on the signal s(t) and window width ω (see Eq. 10).

$$spectrogram\left(t,\omega \right)={\left|STFT\left(t,\omega \right)\right|}^{2}$$ (1)

The spectrogram of a 20 min FHR signal from the DB used in this work is shown in Figure 3G.

### 2.7 Persistence spectrum

The persistence spectrum of a signal is a time-frequency representation that shows the percentage of time a given frequency is present in a signal.

The persistence spectrum is a histogram in power-frequency space. The longer a particular frequency persists in a signal as it evolves, the higher its time percentage and thus the brighter or “hotter” its color in the display.

The calculation of the persistence spectrum is obtained by first computing the spectrogram for a time segment. After that, power and frequency values are partitioned into 2-D bins. For each time value, a bivariate histogram of the logarithm of the power spectrum is computed. For every power-frequency bin where there is signal energy at that instant the corresponding matrix element is increased by one. The persistence spectrum is obtained through the computation of the sum of all the histograms related to every time value. The image obtained presents the Frequency (Hz) on the *x*-axis and the Power Spectrum (dB) on the *y*-axis. Figure 3H shows an example of Persistence Spectrum extracted from an FHR signal belonging to the dataset used in our study.

### 2.8 Neural network architecture

As reported at the beginning of the Methods section, the core idea was to design a neural network capable of dealing with heterogenous data, i.e., a set of scalar values summarizing a signal processing pipeline and a set of images which represent the whole FHR signal in different domains (time-frequency, recurrent periodicities).

Our aim is to integrate the information automatically grasped by two connected branches, each of which is provided with a different kind of input (i.e., images and arrays of values).

This type of approach combines parameters already known to provide information about the physiological mechanisms responsible of the FHR signal, with other characteristics obtained from an implicit understanding made by the model itself. More precisely, the network we designed was fed with an array of 15 quantitative regressors and a set of images, obtained from each FHR sequence of 2400 samples (20 min length, as reported in the previous sections).

The proposed neural architecture includes two branches: an MLP and a CNN. Our purpose was to exploit the ability of CNNs to automatically extract useful features from image data to enrich the information provided by the set of quantitative parameters fed to the MLP branch, which is the most employed architecture when dealing with static data.

The final model was obtained after testing different combinations of layers and parameters for both MLP and CNN branches. We decided to bring together the MLP and CNN branches that individually provided the best results in terms of classification accuracy.

The specifics for the combined CNN + MLP model are listed below.

### 2.9 MLP branch

Input layer: composed of 15 neurons, one for each quantitative parameter passed in input. These neurons are fully connected to the ones of the first hidden layer.

Hidden layer 1: Composed of 500 neurons with ReLU activation function, followed by a Dropout layer with a probability of 0.4, to avoid overfitting.

Hidden layer 2: Composed of 250 neurons with ReLU activation function, followed by a Dropout layer with a probability of 0.4.

Hidden layer 3: Composed of 150 neurons, with ReLU activation function. L1 and L2 regularization penalty is applied. The value for L1 is set to 10–5, for L2 is 10–4.

Hidden layer 4: Composed of 50 neurons, with ReLU activation function followed by a Dropout layer with a probability of 0.4.

Output layer: The 50 nodes of the fourth hidden layer are fully connected to the 2 last neurons of the output layer, with Softmax activation function.

### 2.10 CNN branch

Input layer: The CNN input layer receives as input an array 64 × 64 × 1 x n, where n stands for the number of images fed to the net. The array is created by concatenating n images on the fourth dimension, with n = 1 … 8. The images building up the input array are the ones described in Section 2.4.

Convolutional 2D Layer: The input layer nodes are convoluted by using 16 filters of 5 × 5 kernel, with no padding and ReLU activation function. A Batch Normalization layer is then used to re-scale and recenter the input layer to make the network more stable and faster.

Max Pooling 2D Layer: The first convolutional layer is followed by a Max Pooling Layer with pool size 2 × 2. The pooling operation reduces the eigenarrays of the convolution output and the number of parameters, so it can lower the model complexity and speed up the computation while preventing overfitting.

Convolutional 2D Layer: The second convolutional layer is formed by 32 filters with 5 × 5 kernels, ReLU activation function and no padding, followed by a Batch Normalization layer.

Max Pooling 2D Layer: After the second convolutional layer, a Max pooling Layer with pool size 2 × 2 is added. Dropout is applied with probability 0.8.

Flatten Layer: To unroll the output of the convolutional layers, a Flatten layer is applied.

Dense Layer: Each neuron of the Flatten Layer is fully connected to the 64 neurons of the successive Dense Layer with ReLU activation function, followed by a Batch Normalization layer. A Dropout with 0.8 rate is then applied.

Dense Layer: The 16 neurons with ReLU activation function are fully connected to the last 2 neurons of the output layer.

Output Layer: Consists of 2 neurons, one per class, with Softmax activation function.

### 2.11 Concatenation of MLP and CNN branches

The outputs of MLP and CNN branches are then concatenated to form a single output array passed to the subsequent fully connected layers, through a concatenation layer. From a structural viewpoint, the terminal neurons of MLP and CNN branches are connected to form a flatten layer so that the input to the final set of layers is the output of the layer where MLP and CNN branches are concatenated. This one is followed by a Dense layer of 128 neurons with ReLU activation function. The nodes of the Dense layer are then fully connected to each of the 2 neurons of the output layer, which use a Softmax activation function. These ones give back the probability of the input passed to the artificial network belonging to one of the 2 possible classes (healthy or unhealthy fetus).

### 2.12 Training and testing

The dataset used to train and test the performances of our proposed neural classifier consists of 14′000 labelled examples, of which 7′000 correspond to healthy fetuses and 7′000 to pathological ones. Each example consists of a set of 15 quantitative parameters and a group of images.

80% of the dataset (i.e., 11′200 data) was used to train the neural network, while 20% (i.e., 2800 data) was used for testing the performances of the trained net.

The neural model setup was carried out by using Python 3.7 and for the training phase the online virtual machines provided by (Kaggle, 2021) were used (https://www.kaggle.com/).

We adopted the Adam optimizer with a learning rate of 10–4 and a decay rate of 10–4/200 for the training of the network.

**FIGURE 4:**
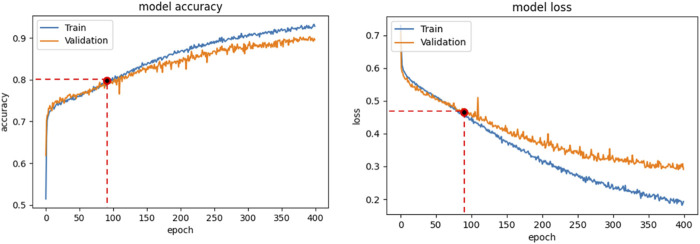
CNN + MLP model: Accuracy curve on training and validation set vs epochs (a); loss curve on training and validation set vs epochs.

Binary-cross-entropy was designed as the loss function to be optimized. Early Stopping technique, with a patience of 2, was employed as an overfitting prevention technique. We always analyzed the relation between the accuracy and loss curves obtained in the different training sessions, in order to verify that overfitting was not occurring. For example, Figure 4 shows the accuracy (a) and loss trends (b) on the training examples for the CNN + MLP net, as functions of the epochs, compared with the accuracy and loss curves on the validation examples. The use of Early stopping, interrupts the training phase at the 90th epoch, preventing the model from excessively adapting to the training data. The crossing point of the red dashed lines in both diagrams of Figure 4 identifies the point where the training is interrupted by the stop criterion.

## 3 Results

As reported in the methods section, we separately evaluated the performance of MLP and CNN branches and compared them with the ones obtained by the CNN + MLP mixed model, in order to state if the latter overperformed respect to MLP and CNN branches singularly.

To provide robustness to the analysis, we repeated the training process 30 times for each of the considered nets. After each training phase was completed, accuracy (ACC), sensitivity (TPR), specificity (TNR), precision (PPV), negative predictive value (NPV), false positive rate (FPR), false negative rate (FNR), false discovery rate (FDR) and Area Under the ROC Curve (AUC) were computed.

The definition of these metrics is reported in Supplementary Appendix SE.

To infer significative statistical differences in terms of average classification accuracy, between the three architectures proposed, T-test was applied.

### 3.1 MLP branch

The MLP branch was fed in every trial with the selected set of 15 features, as described in Section 2.3. The mean accuracy reached by the single MLP, over the 30 train trials, on the 2800 test examples, was 75.5%, i.e., 2115 correct classifications against 685 misclassifications.

The average confusion metrics for the MLP branch is reported in Table 1.

**TABLE 1:** Confusion Matrix for the MLP, CNN, CNN + MLP models obtained on the 2800 examples of test. TP = True Positive, TN = True Negative, FN = False Negative, FP = False Positive.

	MLP		CNN		CNN + MLP	
	Predicted pathological	Predicted healthy	Predicted pathological	Predicted healthy	Predicted pathological	Predicted healthy
True pathological	TP = 998	FN = 427	TP = 681	FN = 596	TP = 960	FN = 431
True healthy	FP = 258	TN = 1117	FP = 298	TN = 1225	FP = 109	TN = 1300

The average values, for the different performance metrics computed for the model, are instead summarized in Table 2.

**TABLE 2:** Performance metrics for the MLP, CNN, CNN + MLP models. It reports: True Positive Rate [TPR = TP/(TP + FN)], even called Recall or Sensitivity, True Negative Rate [TNR = TN/(TN + FP)] or Specificity, Positive Predictive Value [PPV = TP/(TP + FP)] or precision, Negative predictive value [NPV = TP/(TP + FN)], fall out or false positive rate [FPR = FP/(FP + TN)], False negative rate [FNR = FN/(TP + FN)], False discovery rate [FDR = FP/(TP + FP)].

	Performance metrics							
	TPR	TNR	PPV	NPV	FPR	FNR	FDR	AUC
MLP	0.7	0.81	0.79	0.72	0.18	0.29	0.2	0.76
CNN	0.53	0.80	0.69	0.53	0.19	0.46	0.3	0.67
CNN + MLP	0.69	0.92	0.90	0.75	0.08	0.31	0.1	0.81

### 3.2 CNN branch

For what concerns the CNN branch, we firstly had to choose which combination of images (see methods Section 2.4) provided as input to the net, could lead to the best results. For that aim, we tested all the different combinations of images, and for each of them we trained the CNN a number of 15 times, computing the classification accuracy at each step.

At the end of the process, we selected the combination of images providing in average the highest accuracy. By looking at the results achieved we can state that the most impactful images are GADF, followed by PS and PSP.

After selecting the most performing CNN architecture, we trained the latter a number of 30 times and for each phase we computed all the performance metrics, whose mean values are summarized in Table 2. The overall accuracy obtained is 68.1%, i.e., 1906 correct classifications against 894 misclassifications. The confusion matrix for the CNN branch is summarized in Table 1.

### 3.3 Combined model (CNN + MLP)

After evaluating MLP and CNN branches separately, we tested the performances of the combined CNN + MLP model, that concatenates MLP and CNN nets in a single mixed architecture.

As for the single CNN case, we had to select the top performing combination of images to feed the CNN branch of the combined model. Even in this case, the most impactful images have been proved to be GADF, PS and PSP.

After selecting the most suitable inputs for the CNN branch of the combined model, we repeated the training phase of the CNN + MLP net 30 times.

The confusion matrix obtained for the trained CNN + MLP net on the 2800 data composing the test set is shown in Table 1, while the average performance metrics obtained with the best CNN + MLP model are summarized in Table 2.

### 3.4 Comparison between MLP, CNN and CNN + MLP models

**FIGURE 5:**
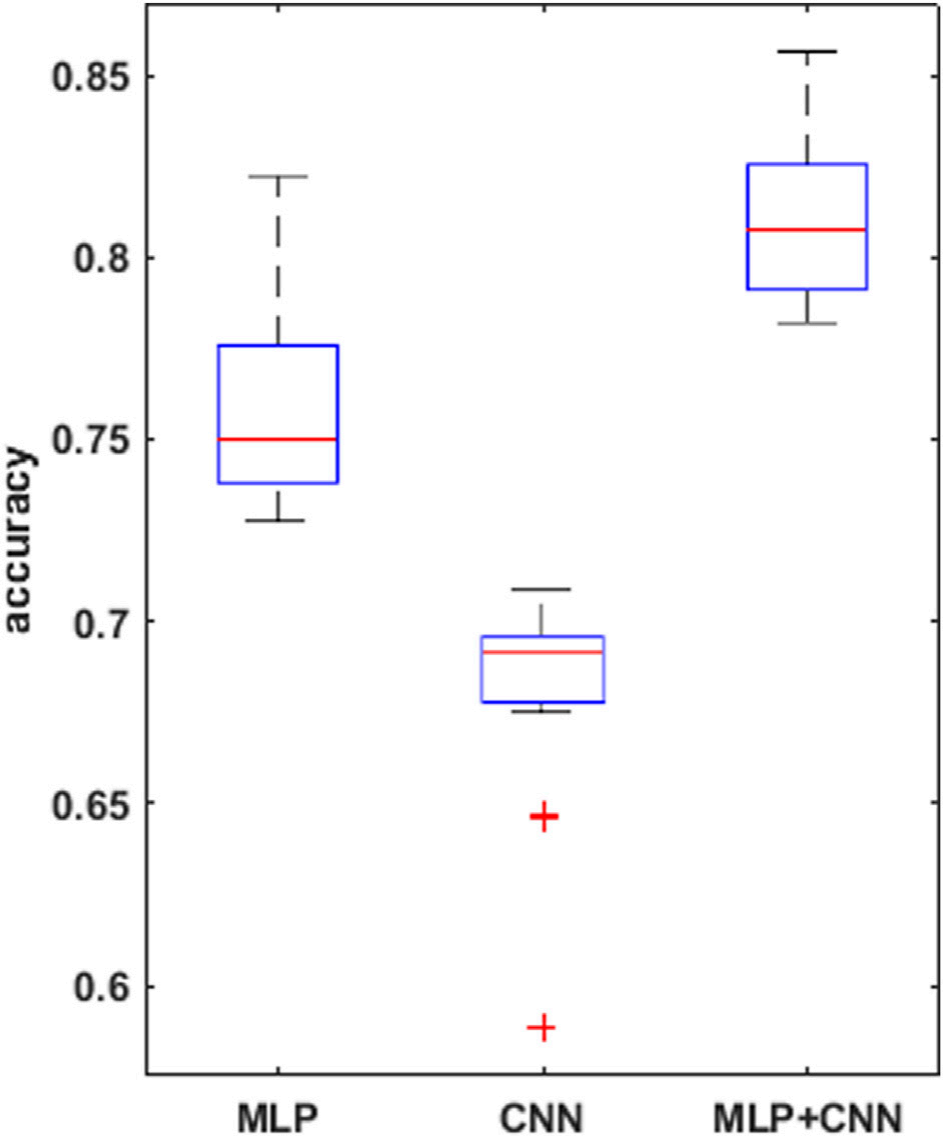
boxplots for the mean accuracy values reached, over the 30 replications of the training phase, for the 3 models compared, i.e., MLP, CNN, combined CNN + MLP.

**FIGURE 6:**
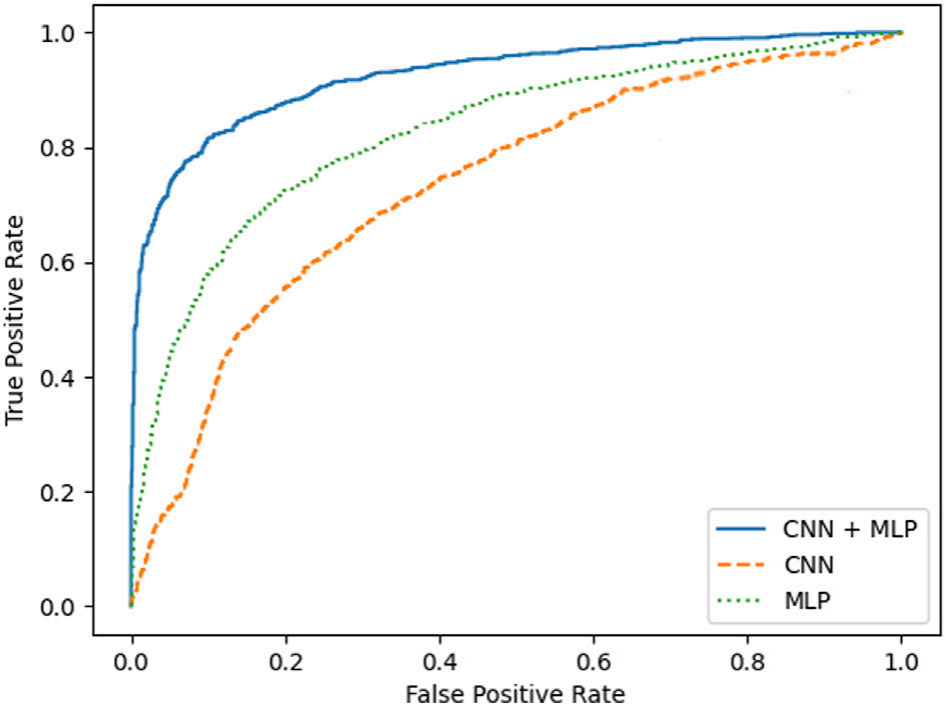
ROC curves for MLP, CNN and CNN + MLP combined neural model developed.

A summary of the overall accuracy achieved by the different models evaluated is reported in Table 3, while the corresponding boxplot and ROC curves are illustrated in Figures 5, 6 respectively.

**TABLE 3:** Summary of overall accuracy achieved for the MLP, CNN and CNN + MLP models.

	Mean accuracy (%)	Number of correct classifications	Number of wrong classifications
MLP	75.7	2120	680
CNN	68.1	1907	893
CNN + MLP	**80.1**	**2260**	**540**

To prove significative statistical differences among the three nets explored, T-test was applied to challenge the null hypothesis (H0) of equality, in terms of average classification accuracy, between the three models. With a level of significance of 0.01, H0 was refused for every comparison performed.

From the observation of the obtained results, it appears how the use of the convolutional branch alone does not allow to reach an adequate classification accuracy, showing lower performances than those obtained with the single MLP branch. However, the results achieved with the combined CNN + MLP model show a significant increase in the classification capacity of the model, compared to the MLP and CNN architectures individually considered. The combined CNN + MLP model, in fact, reached an overall classification accuracy of 80.1%. This corresponds to a total number of 2260 correct classifications against 540 misclassifications.

The combined model proposed, hence, seems to be able to exploit the good accuracy of the MLP to influence and boost the performance of the CNN on the provided images, confirming how the combined use of known quantitative regressors and features, implicitly learnt from the neural model, could increase the classification capabilities.

There is however to point out that the neural model realized tends to better classify the signals related to healthy fetuses (FP = 109, FN = 431). In fact, the CNN-MLP model presents a high specificity (TNR) of 92%, but its sensitivity (TPR) is of 69%. This means that the proposed architecture misclassifies a signal related to a healthy subject the 8% of times while misses the classification 31% of times when dealing with a signal referred to an unhealthy subject.

## 4 Conclusion

The possibility to identify early signs of fetal sufferance antepartum still remains a dream in the Ob-Gyn management of pregnancies. An accurate disambiguation between healthy and suffering fetuses can allow obstetricians to intervene in a timely manner and take appropriate actions to prevent permanent damages to the fetus. Among the prenatal exams, the CTG represents the major source of information on the correct development of the fetus.

Despite the fast increase of the digital technology in medical devices, in the clinical practice, the analysis of CTG signals, both antepartum and during labor, is mostly carried out by visual analysis of the tracings. This procedure is obviously affected by significant inter-observer and intra-observer variability, which often causes erroneous interpretations of real fetal conditions.

The introduction of computerized CTG analysis decreased the qualitative and subjective interpretation of the CTG exam, but didn’t lead to a reliable clinical decision-making strategy, despite the great effort produced in the past 20 years for extracting significant quantitative indices from the FHR signal.

Artificial Intelligence techniques, with a particular focus on Deep Learning, represent a further tool to investigate the information content of CTG tracings, although they need huge datasets in order to provide reliable conclusions. As we had available a considerable amount of annotated CTG exams, we decided to approach the problem of classifying normal and pathological fetuses by means of those methods.

The availability of a very large and structured database, consisting of real labeled data that were collected in the same clinical department, represents the first important aspect of this work. This feature is difficult to find in the field of fetal monitoring. It has made possible to exploit machine learning and deep learning methods to the best of their abilities. In fact, it is known that the classification power of AI methods is best expressed only with large amounts of data, which was not allowed until now for the analysis of the fetal heart variability signal.

A second factor is the correspondence between the quantitative values of the parameters used for classification and the fetal and maternal physiology. Each parameter we have employed (and the 15-feature set is an example), can contribute to the understanding of the physiological mechanisms that controls fetal heart. These features make readable and interpretable the data set in terms of control developed by physiological systems.

The classification proposed in this paper benefits from the information contained in these parameters. Therefore, it is possible to formulate a classification between healthy and pathological fetuses that is interpretable according to involved pathophysiology, whose measurements take place through the parameters extracted in the FHR.

We designed and implemented a neural architecture able to deal with heterogeneous data, i.e., images and quantitative parameters describing the statistical characteristics of the FHR signal. The neural network consists of two branches, a MLP receiving an array of 15 regressors and a CNN one fed with a set of 64 × 64 images. The latter have been obtained through several transformations (e.g., MTF, GADF, RP, etc.) applied to the pre-processed denoised FHR signal.

To understand if the novel mixed-type architecture overperforms the MLP and CNN branches singularly, we compared the results obtained from the three neural architectures in terms of overall classification accuracy. After the hyperparameters’ optimization for each NNs, the MLP, CNN and MLP-CNN architecture have been trained and tested on a set of 14 K data (split in 80% for the training and 20% for the test). The results obtained have shown that the MLP-CNN network is the best performing architecture. Hence, with the best set of hyperparameters, this mixed-type net achieved an overall classification accuracy of 80.1%.

The major limitation of the Method still lies in the sensitivity, which is not yet fully satisfactory. In fact, with the combined model (CNN + MLP) the TPR reached is of 69%, that corresponds to a probability of erroneous classification of an unhealthy subject of 31%.

There is, however, to consider that unhealthy subjects contained in the database and used for this work, are a heterogeneous group, which includes several types of diseases: intra uterine growth restriction (IUGR), metabolic alterations, fetal malformations, and even maternal pathological conditions, such as diabetes.

The decision to include all the different categories of disease in the unhealthy class made it possible to balance the number of healthy and unhealthy subjects with a sufficient numerosity to allow the use of Deep Learning techniques. This could lower the performance of our neural model, both in terms of accuracy and specificity, since different pathologies could show different behaviors in the FHR signals, reducing the classification capacities and increasing the variability of FHR features. These analyses must be considered as a starting point in the direction of more complex studies, that look at the different classes of pathologies separately, once the amount of data for each pathology will reach an acceptable value for Deep Learning methods.

Nevertheless, the obtained results are promising, since they have been achieved by using a noticeable amount of clinical data, whose variability closely represents the real population. Although this fact may reduce the classification performance, as compared to other existing works, it can however increase the robustness and the generalization ability of the model.

Further developments for this work include the search for other techniques for converting the CTG signals into images, in order to provide new kinds of inputs to the CNN branch. In addition, more quantitative parameters, to feed the MLP branch, will be investigated. Moreover, other mixed-type neural architectures will be explored and will include other types of neural branches such as Recurrent NN or Temporal CNN.

## Data Availability

The datasets presented in this article are not readily available because Subsets of data can be available under request to the corresponding author for research purposes with the commitment to mention the source. Requests to access the datasets should be directed to GM, giovanni.magenes@unipv.it.
