# Supplementary material for: A deep learning mixed-data type approach for the classification of FHR signals
Source: Front Bioeng Biotechnol. 2022 Aug 8;10:887549. doi: 10.3389/fbioe.2022.887549 (PMC9393210; doi:10.3389/fbioe.2022.887549)
Supplement: Supplementary file 1 [file DataSheet2.PDF]

Tables

Table 1: Best MLP architecture tested with relating parameters. It contains the number of neurons employed for the nth layer Ln (n = 1…4) with ReLU activation function, the dropout rate (D) for the layer Ln, the optimizer (O) used, the learning rate (LR), the decay rate (DR), and the mean accuracy (ACC) over ten trials.

Table 1- Best 10 MLP Models													
MLP Models	Layers and parameters												
		**L1**	**D**	**L 2**	**D**	**L 3**	**D**	**L 4**	**D**	**O**	**LR**	**DR**	**ACC**
	**Model 1**	50	0.4	150	0.4	250	0.4	500	x	Adam	10^{-5}	$10^{-4/100}$	**69.2%**
	**Model 2**	50	0.4	150	0.4	250	0.4	500	x	Adam	10^{-5}	$10^{-4/200}$	**70.1%**
	**Model 3**	500	0.4	250	0.8	150	x	50	0.8	Adam	10^{-4}	$10^{-4/100}$	**71.3 %**
	**Model 4**	300	x	500	x	150	x	x	x	Adam	10^{-4}	0	**72.0 %**
	**Model 5**	500	0.4	250	0.4	150	0.4	50	0.4	Adam	10^{-3}	$10^{-4/100}$	**72.8 %**
	**Model 6**	500	0.4	250	0.6	150	x	50	0.9	Adam	10^{-4}	$10^{-4/100}$	**74.3 %**
	**Model 7**	500	0.4	250	0.4	150	0.4	x	x	Adam	10^{-4}	$10^{-4/100}$	**74.8 %**
	**Model 8**	500	0.4	250	0.4	150	0.4	x	x	Adam	10^{-4}	$10^{-4/100}$	**75.3 %**
	**Model 9**	500	x	300	x	150	x	x	x	Adam	10^{-4}	$10^{-4/100}$	**75.4 %**
	**Model 10**	**500**	**0.4**	**250**	**0.4**	**150**	**x**	**50**	**x**	**Adam**	$\mathbf{10^{-4}}$	$\mathbf{10^{-4/100}}$	**75.7 %**

Table 2: Best 10 CNN branch architectures and relating parameters tested**.** It reports the number of filters (f) and the kernel size (k) for each 2-dimensional CNN layer, the presence or absence of Batch Normalization layers (BN), the pool size of each Max Pooling layer (MP), the number of neurons employed for the nth Dense layer with ReLU activation function, the dropout rate (D) for the layer L n (n = 1…5), the optimizer (O) used, the learning rate (LR), the decay rate (DR), and the mean accuracy (ACC) over ten trials. Layers 1, 2 and 3 are 2D-CNN layers while Layers 4 and 5 are Dense ones.

	Table 2 - Best 10 CNN Models
	CNN models

Layers and parameters		Model 1	Model 2	Model 3	Model 4	Model 5	Model 6	Model 7	Model 8	Model 9	Model 10
	**f**	16	16	8	8	32	16	16	16	16	**16**
	**k**	5	5	5	5	5	3	3	3	5	**5**
	**L1**	f, k	f, k	f, k	f, k	f, k	f, k	2f, k	2f, k	2f, k	**2f, k**
	**B**	v	v	v	v	v	v	v	v	v	**v**
	**M**	2	2	2	2	2	2	2	2	2	**2**
	**D**	0	0	0.4	0.7	0.5	0.8	0.6	0.4	0.8	**x**
	**L2**	2f, k	2f, k	2f, k	2f, k	2f, k	f, k	2f, k	2f, k	f, k	**2f, k**
	**B**	v	v	v	v	v	v	v	v	v	**v**
	**M**	2	2	2	2	2	2	2	2	2	**2**
	**D**	0.8	0.8	0.4	0.6	0.5	0.3	0	0	0	**0.8**
	**L3**	x	x	x	x	x	x	x	x	x	**x**
	**M**	x	x	x	x	x	x	x	x	x	**x**
	**L4**	4	4	4	4	4	4	4	4	4	**64**
	**L5**	x	x	x	x	x	x	x	x	x	**16**
	**O**	Adam	Adam	Adam	Adam	Adam	Adam	Adam	Adam	Adam	**Adam**
	**LR**	10^{-4}	10^{-4}	10^{-4}	10^{-4}	10^{-5}	10^{-5}	10^{-4}	10^{-5}	10^{-4}	$\mathbf{10^{-4}}$
	**DR**	0	$10^{-4/100}$	$10^{-4/100}$	$10^{-4/100}$	$10^{-4/100}$	$10^{-4/200}$	$10^{-4/100}$	$10^{-4/100}$	$10^{-4/200}$	$\mathbf{10^{-4/200}}$
	**ACC**	**65.1%**	**65.3%**	**65.7%**	**65.9%**	**66.4%**	**66.7%**	**66.8%**	**67.0%**	**67.2%**	**68.1%**

Table 3*: Confusion Matrix for the MLP model. TP = True Positive, TN = True Negative, FN = False Negative, FP =False Positive.*

Table 3 - Confusion matrix for the MLP

	Predicted pathological	Predicted healthy
**True pathological**	TP = 998	FN = 427
**True healthy**	FP = 258	TN = 1117

Table 4: *Performance metrics for the MLP model. It reports : True Positive Rate (TPR = TP/(TP+FN)), even called Recall or Sensitivity, True Negative Rate (TNR = TN/(TN+FP)) or Specificity, Positive Predictive Value (PPV = TP/ (TP + FP)) or precision, Negative predictive value (NPV = TP/ (TP + FN)), Fall out or false positive rate (FPR = FP/ (FP + TN)), False negative rate (FNR = FN/ (TP + FN)), False discovery rate (FDR = FP/ (TP + FP)).*

**Table 4: - Performance metrics for the MLP**						
**TPR**	**TNR**	**PPV**	**NPV**	**FPR**	**FNR**	**FDR**
0.7	0.81	0.79	0.72	0.18	0.29	0.2

Table 5*: Mean accuracy for the different combinations of images with CNN. A value of 0 in Table 3 means the exclusion of the image while a value of 1 means its inclusion.*

**Table 5 - Best 10 combinations of images for the CNN**								
**CWT**	**GADF**	**GASF**	**MTF**	**RP**	**S**	**PS**	**PSP**	**ACC**
**0**	**1**	**0**	**0**	**0**	**0**	**1**	**1**	**68.1%**
1	1	1	1	1	0	1	1	67.7%
1	1	1	1	1	1	1	0	67.5%
0	1	0	0	0	0	0	1	67.0%
1	1	0	1	0	1	1	0	66.9%
1	1	1	1	1	0	1	0	66.4%
0	1	0	0	0	1	1	0	66.3%
0	1	0	0	0	1	0	1	66.3%
0	1	0	0	0	0	0	1	66.2%

1	1	0	1	0	1	0	1	65.9%

Table 6 *Performance metrics for the MLP model. It reports : True Positive Rate (TPR = TP/(TP+FN)), even called Recall or Sensitivity, True Negative Rate (TNR = TN/(TN+FP)) or Specificity, Positive Predictive Value (PPV = TP/ (TP + FP)) or precision, Negative predictive value (NPV = TP/ (TP + FN)), Fall out or false positive rate (FPR = FP/ (FP + TN)), False negative rate (FNR = FN/ (TP + FN)), False discovery rate (FDR = FP/ (TP + FP)).*

**Table 6 - Performance metrics for the CNN**						
**TPR**	**TNR**	**PPV**	**NPV**	**FPR**	**FNR**	**FDR**
0.53	0.80	0.69	0.53	0.19	0.46	0.3

Table 7*: Confusion Matrix for the CNN model. TP = True Positive, TN = True Negative, FN = False Negative, FP =False Positive.*

**Table 7 - Confusion matrix for the CNN**		
	**Predicted pathological**	**Predicted healthy**
**True pathological**	TP = 681	FN = 596
**True healthy**	FP = 298	TN = 1225

Table 8*: Mean accuracy for the different combinations of images with CNN+MLP. A value of 0 in Table 3 means the exclusion of the image while a value of 1 means its inclusion.*

**Table 8 - Best 10 combinations of images for the CNN+MLP**								
**CWT**	**GADF**	**GASF**	**MTF**	**RP**	**S**	**PS**	**PSP**	**ACC**
**0**	**1**	**0**	**0**	**0**	**0**	**1**	**1**	**80.1%**
1	1	1	1	1	0	1	1	78.6%
1	1	1	1	1	1	1	0	78.4%
0	1	0	0	0	0	0	1	78.4%
1	1	0	1	0	1	1	0	78.4%
1	1	1	1	1	0	1	0	78.3%

0	1	0	0	0	1	1	0	78.3%
0	1	0	0	0	1	0	1	77.9%
0	1	0	0	0	0	0	1	77.9%
1	1	0	1	0	1	0	1	77.8%

Table 9*: Confusion Matrix for the CNN+MLP model. TP = True Positive, TN = True Negative, FN = False Negative, FP =False Positive.*

Table 9 - Confusion matrix for the CNN+MLP		
	**Predicted pathological**	**Predicted healthy**
**True pathological**	TP = 960	FN = 431
**True healthy**	FP = 109	TN = 1300

Table 10: *Performance metrics for the CNN+MLP model. It reports : True Positive Rate (TPR = TP/(TP+FN)), even called Recall or Sensitivity, True Negative Rate (TNR = TN/(TN+FP)) or Specificity, Positive Predictive Value (PPV = TP/ (TP + FP)) or precision, Negative predictive value (NPV = TP/ (TP + FN)), Fall out or false positive rate (FPR = FP/ (FP + TN)), False negative rate (FNR = FN/ (TP + FN)), False discovery rate (FDR = FP/ (TP + FP)).*

Table 10 - Performance metrics for the CNN+MLP						
**TPR**	**TNR**	**PPV**	**NPV**	**FPR**	**FNR**	**FDR**
0.69	0.92	0.90	0.75	0.08	0.31	0.1

Table 11*: Summary of overall accuracy achieved within 10 trials for the MLP, CNN and MLP+CNN models.*

Table 11 - Mean classification accuracy			
**Neural Network**	**Mean accuracy**	**Number of correct classifications**	**Number of wrong classifications**
**MLP**	75.7%	2120	680
**CNN**	68.1%	1907	893

**MLP+CNN**	**80.1%**	**2260**	**540**
