# Supplementary material for: A deep learning mixed-data type approach for the classification of FHR signals
Source: Front Bioeng Biotechnol. 2022 Aug 8;10:887549. doi: 10.3389/fbioe.2022.887549 (PMC9393210; doi:10.3389/fbioe.2022.887549)
Supplement: Supplementary file 2 [file DataSheet3.docx]

**Supplementary Appendix**

1. **Continuous wavelet transform (CWT)**

The CWT is defined as the summation of the overall signal spectrum multiplied by the compressed and translated “mother wavelet”, which can be expressed mathematically by the following equations:

$\boldsymbol{CWT}\left( \boldsymbol{\tau,s} \right)\boldsymbol{=\psi}\left( \boldsymbol{\tau,s} \right)\boldsymbol{=}\int_{\boldsymbol{-\infty}}^{\boldsymbol{\infty}} \boldsymbol{f(t)}\boldsymbol{\phi}_{\boldsymbol{\tau}}^{\boldsymbol{*}}$ $\boldsymbol{f}\left( \boldsymbol{t} \right)\boldsymbol{\epsilon}\boldsymbol{L}^{\boldsymbol{2}}\boldsymbol{(R)}$ (2)

$\boldsymbol{\phi}_{\boldsymbol{\tau,s}}\left( \boldsymbol{t} \right)\boldsymbol{=}\frac{\boldsymbol{1}}{\sqrt{\left| \boldsymbol{S} \right|}}\boldsymbol{\phi}\left( \frac{\boldsymbol{t-\tau}}{\boldsymbol{s}} \right)\boldsymbol{\tau,s \epsilon R, s\neq0}$ (3)

$\int_{\boldsymbol{-\infty}}^{\boldsymbol{+\infty}} \boldsymbol{\phi}\left( \boldsymbol{t} \right)\boldsymbol{ⅆt}\boldsymbol{=0}$ (4)

In equation (2) *f(t)* represents the input signal, *φ(t)* is wavelet basis, and *ψ (τ, s)* represents the wavelet coefficient, a function of two variables. *τ* is the translation factor that reflects temporal and spatial information and represents the translation diameter of time shifting; *s* is the scaling factor that determines the degree to which the wavelet is compressed or stretched.

1. **Gramian angular field**

First, let’s recall that the dot product (or inner product) is a measure of similarity between two arrays. I.e., if we consider two vectors (*x*_1_ and *x*_2_), the dot product between *x*_1_ and *x*_2_ is defined as follows:

$\left\langle\boldsymbol{x}_{\boldsymbol{1}}\boldsymbol{,}\boldsymbol{x}_{\boldsymbol{2}} \right\rangle\boldsymbol{=}\left\| \boldsymbol{x}_{\boldsymbol{1}} \right\|\left\| \boldsymbol{x}_{\boldsymbol{2}} \right\|\cos\boldsymbol{\theta}$ (5)

where *θ* is the angle between *x*_1_ and *x*_2_. If we don’t consider the magnitude of the arrays, we can state that if the angle between *x*_1_ and *x*_2_ is small (i.e., close to 0) then the cosine of that angle will be nearly 1. If *x*_1_ and *x*_2_ are perpendicular, the cosine of the angle is 0. If the two vectors are pointing in opposite directions, the cosine will be -1. Starting from this consideration it is possible to obtain the Gram Matrix *G* as:

$\boldsymbol{G=}\left( \begin{matrix} \left\langle\boldsymbol{x}_{\boldsymbol{1}}\boldsymbol{,}\boldsymbol{x}_{\boldsymbol{1}} \right\rangle& \boldsymbol{\cdots} & \left\langle\boldsymbol{x}_{\boldsymbol{1}}\boldsymbol{,}\boldsymbol{x}_{\boldsymbol{N}} \right\rangle\\ \boldsymbol{\vdots} & \boldsymbol{\ddots} & \boldsymbol{\vdots} \\ \left\langle\boldsymbol{x}_{\boldsymbol{N}}\boldsymbol{,}\boldsymbol{x}_{\boldsymbol{1}} \right\rangle& \boldsymbol{\cdots} & \left\langle\boldsymbol{x}_{\boldsymbol{N}}\boldsymbol{,}\boldsymbol{x}_{\boldsymbol{N}} \right\rangle\end{matrix} \right)$ (6)

Gramian Angular Field (GAF) is obtained by introducing a “special” inner product as follows.

Let’s suppose we are given a time series *X= {x*_1_*, x*_2_*, …, x*_N_*}*, normalized to be in [-1,1]. The first step is to convert each value in *X* into polar coordinates, through the transformation:

$\boldsymbol{\phi}_{\dot{\boldsymbol{i}}}\boldsymbol{=}\arccos\left( \boldsymbol{x}_{\boldsymbol{i}} \right)$ (7)

Finally, the GAF method defines its “special” inner product as:

$\left\langle\boldsymbol{x}_{\boldsymbol{i}}\boldsymbol{,}\boldsymbol{x}_{\boldsymbol{j}} \right\rangle\boldsymbol{=}\cos\left( \boldsymbol{\phi}_{\boldsymbol{i}}\boldsymbol{+\phi j} \right)$ (8)

to obtain GASF, and:

$\left\langle\boldsymbol{x}_{\boldsymbol{i}}\boldsymbol{,}\boldsymbol{x}_{\boldsymbol{j}} \right\rangle\boldsymbol{=}\sin\left( \boldsymbol{\phi}_{\boldsymbol{i}}\boldsymbol{-}\boldsymbol{\phi}_{\boldsymbol{j}} \right)$ (9)

to obtain GADF.

1. **Markov transition field**

The Markov transition field (MTF) is another way to convert a time series into an image. Given a time series *X*= {*x*_1_*, x*_2_*, …, x*_n_}, a data point *x_i_* is assigned to its corresponding quantile bin *q_j_ (1≤ j ≤Q)* where *Q* is the number of bins, i.e., the number of states. In this way we can construct, from *X,* a Markov chain, deriving the *Q x Q* Markov transition matrix (*W*) where *w_ij_ (1≤ I, j ≤Q)* in *W* is the frequency with which a data point in the state q_j_ is followed by a data point in state *q_i_*. After normalization by $\sum_{j} w_{ij}$ = 1, *W* is the Markov Transition Matrix, where *w_ij_* represents the transition probability of *q_i_ → q_j_* in the MTF. By assigning the probability from the quantile at time step *i* to the quantile at time step *j* at each pixel *M_ij_*, the MTF, denoted as *M* (see equation (7)), encodes the multi-span transition probabilities of the time series. The main diagonal *M_ii_* captures the probability from each quantile to itself (the self-transition probability) at time step *i*.

$\boldsymbol{M=}\left[ \begin{matrix} \boldsymbol{w}_{\boldsymbol{ij}}\boldsymbol{|}\boldsymbol{x}_{\boldsymbol{1}}\boldsymbol{\epsilon}\boldsymbol{q}_{\boldsymbol{i}}\boldsymbol{,}\boldsymbol{x}_{\boldsymbol{1}}\boldsymbol{\epsilon}\boldsymbol{q}_{\boldsymbol{j}} & \boldsymbol{\cdots} & \boldsymbol{w}_{\boldsymbol{ij}}\boldsymbol{|}\boldsymbol{x}_{\boldsymbol{1}}\boldsymbol{\epsilon}\boldsymbol{q}_{\boldsymbol{i}}\boldsymbol{,}\boldsymbol{x}_{\boldsymbol{n}}\boldsymbol{\epsilon}\boldsymbol{q}_{\boldsymbol{j}} \\ \boldsymbol{\vdots} & \boldsymbol{\ddots} & \boldsymbol{\vdots} \\ \boldsymbol{w}_{\boldsymbol{ij}}\boldsymbol{|}\boldsymbol{x}_{\boldsymbol{1}}\boldsymbol{\epsilon}\boldsymbol{q}_{\boldsymbol{i}}\boldsymbol{,}\boldsymbol{x}_{\boldsymbol{1}}\boldsymbol{\epsilon}\boldsymbol{q}_{\boldsymbol{j}} & \boldsymbol{\cdots} & \boldsymbol{w}_{\boldsymbol{ij}}\boldsymbol{|}\boldsymbol{x}_{\boldsymbol{1}}\boldsymbol{\epsilon}\boldsymbol{q}_{\boldsymbol{i}}\boldsymbol{,}\boldsymbol{x}_{\boldsymbol{n}}\boldsymbol{\epsilon}\boldsymbol{q}_{\boldsymbol{j}} \end{matrix} \right]$ (10)

1. **Recurrence plot**

The RP is defined by matrix *R*, following equation (11)

$\boldsymbol{R}\left( \boldsymbol{i,j} \right)\boldsymbol{=}\left\{ \begin{aligned} \boldsymbol{1 if}\left| \left| \vec{\boldsymbol{x}}\left( \boldsymbol{i} \right)\boldsymbol{-}\vec{\boldsymbol{x}}\left( \boldsymbol{j} \right) \right| \right|\boldsymbol{\leq\epsilon} \\ \boldsymbol{0 otherwise} \end{aligned} \right.$ (11)

$\vec{x}$(i) and $\vec{x}$(j) represent the states, $\|.\|$ is the first norm and ε is a threshold. *R(i,j)* is 1 if $\vec{x}$(i) ≈$\vec{x}$(j) up to an error *ε*. This one is fundamental since systems often do not recur exactly to a previously visited state but tend to visit roughly the same area in the phase space. Similarly, a RP can be generated by first computing a distance matrix *S* that contains each pair of distances and then applying the threshold *ε*, as defined in equation (12)

$\boldsymbol{S=}\left| \left| \vec{\boldsymbol{x}}\left( \boldsymbol{i} \right)\boldsymbol{-}\vec{\boldsymbol{x}}\left( \boldsymbol{j} \right) \right| \right|$ (12)

1. **Performance Metrics**

$$\boldsymbol{Accuracy (ACC)=}\frac{\boldsymbol{TP+TN}}{\boldsymbol{TP+TN+FP+FN}} (13)$$

$$\boldsymbol{True}\boldsymbol{Positive}\boldsymbol{Rate}\left( \boldsymbol{TPR} \right)\boldsymbol{=Recall=Sensitivity=}\frac{\boldsymbol{TP}}{\boldsymbol{TP+FN}} (14)$$

$$\boldsymbol{True Negative Rate}\left( \boldsymbol{TNR} \right)\boldsymbol{=Specificity=}\frac{\boldsymbol{TN}}{\boldsymbol{TN+FP}} (15)$$

$$\boldsymbol{Positive Predictive Value}\left( \boldsymbol{PPV} \right)\boldsymbol{=Precision=}\frac{\boldsymbol{TP}}{\boldsymbol{TP+FP}} (16)$$

$$\boldsymbol{Negative Predictive Value}\left( \boldsymbol{NPV} \right)\boldsymbol{=}\frac{\boldsymbol{TP}}{\boldsymbol{TP+FN}} (17)$$

$$\boldsymbol{False Positive Rate}\left( \boldsymbol{FPR} \right)\boldsymbol{= Fall out=}\frac{\boldsymbol{FP}}{\boldsymbol{FP+TN}} (18)$$

$$\boldsymbol{False Negative Rate}\left( \boldsymbol{FNR} \right)\boldsymbol{=}\frac{\boldsymbol{FN}}{\boldsymbol{TP+FN}} (19)$$

$$\boldsymbol{False Discovery Rate}\left( \boldsymbol{FDR} \right)\boldsymbol{=}\frac{\boldsymbol{FP}}{\boldsymbol{TP+FP}} (20)$$
